# The efficacy and safety of local 5-aminolevulinic acid-based photodynamic therapy in the treatment of cervical high-grade squamous intraepithelial lesion: a single center retrospective observational study

**DOI:** 10.3389/fonc.2024.1390982

**Published:** 2024-04-16

**Authors:** Jing Qian, Yahui Wang, Guihong Wu, Junlei Lu, Liping Sun, Song Xu

**Affiliations:** ^1^ Department of Gynecology, Affiliated Hangzhou First People’s Hospital, Westlake University School of Medicine, Hangzhou, Zhejiang, China; ^2^ Department of Gynecology, TongLu County Maternal and Child Health Hospital, Hangzhou, Zhejiang, China; ^3^ Fourth Clinical School of Medicine, Zhejiang University of Chinese Medicine, Zhejiang, Hangzhou, China

**Keywords:** photodynamic therapy, 5-aminolevulinic acid, cervical high-grade squamous intraepithelial lesion, high-risk HPV, cervical cancer

## Abstract

**Background:**

Typical treatments for cervical high-grade squamous intraepithelial lesion (HSIL) are invasive procedures. However, these procedures often come with several severe side effects, despite their positive effects on cervical HSIL. 5-aminolevulinic acid photodynamic therapy (ALA-PDT) is a non-invasive treatment that has been successfully used to treat cervical low-grade squamous intraepithelial lesion (LSIL). In this study, we aimed to further investigate the clinical efficacy and safety of ALA-PDT in the treatment of patients with cervical HSIL.

**Methods:**

A total of 40 patients aged 20 - 41 years with cervical HSIL and high-risk Human Papilloma Virus (HR-HPV) infections were enrolled in this retrospective study from January 2019 to December 2022. Patients were treated with six times of ALA-PDT at intervals of 7–14 days. Three months after the treatment, the efficacy was evaluated through HPV genotyping and cervical cytology examination. If the cytological result was worse than ASC -US, the patient underwent colposcopy-directed biopsy immediately. Otherwise, patients would receive rigorous follow-up observation.

**Results:**

Three months after receiving ALA-PDT treatment, 65% (26/40) of cervical HSIL patients at our center showed complete regression (cytological result: normal; HR-HPV: negative). This rate increased to 82.5% (33/40) at the 12-month follow-up. None of the patients experienced disease progression after ALA-PDT therapy. The risk of persistent HR-HPV infection was 32.5% (13/40) at the 3-month follow-up after ALA-PDT. Multivariate analyses identified cervical canal involvement as an independent risk factor for persistent HR-HPV infection at the 3-month follow-up after ALA-PDT treatment. During the treatment of the 40 patients with ALA-PDT, there were no reports of severe adverse reactions. Only a limited number of patients experienced slight discomfort symptoms.

**Conclusion:**

ALA-PDT is safe and effective noninvasive therapy for patients with cervical HSIL and HR-HPV infections. It is particularly suitable for young women, who have been confirmed with cervical HSIL and have demand for fertility protection. Three months after ALA-PDT treatment, if a patient still has either ASC-US cervical cytological result and/or HR-HPV infection, rigorous observation is considered safe for her. Cervical canal involvement is an independent risk factor for persistent HR-HPV infection at the 3-month follow-up after ALA-PDT treatment.

## 1 Introduction

Cervical cancer is a highly prevalent and severe life-threatening malignancy among women worldwide. More than 85% of cervical cancer cases take place in developing countries (1). Notably, in China alone, there were approximately 150,700 new cases and 55,700 deaths in 2022 (2). Hence, cervical cancer is a major public health problem affecting women in China. Histologically confirmed cervical HSIL, a precursor to cervical cancer, occurs as a result of persistent HR-HPV infection, has a risk of 20-30% to progress into invasive carcinoma within 10 years (3). Conventionally, managements for cervical HSIL including cold knife conization, laser conization, laser ablation and loop electrosurgical excision procedure (LEEP) (4), which can lead to cervical distortion, excessive destruction of tissue, and subsequent obstetric complications such as preterm labor, premature rupture of amniotic membranes, chorioamnionitis, low birth weight, and increased morbidity in the newborns (5–7). In China, with the gradual liberalization of fertility policies and the younger onset age of the female lower genital tract diseases, there is an increasing demand to protect normal cervical structure and preserve fertility. Therefore, there is a desirable need to explore effective conservative treatments with minimal damage and fewer adverse reactions for managing cervical HSIL.

Photodynamic therapy (PDT) is an emerging alternative technique for the treatment of squamous intraepithelial lesions. It works through the interaction of photosensitizing agents, light and oxygen, providing a non-invasive, effective and targeted treatment (8). 5-aminolevulinic acid (5-ALA), as a second-generation photosensitizer, can accumulate in higher concentrations in pathological cells and absorb light of the appropriate wavelength. This initiates the photodynamic reactions and selective destruction of inappropriate tissues (8, 9). In recent years, several studies have reported the satisfactory efficacy and safety of PDT in treating cervical LSIL (10–13). Chinese experts in gynecology and obstetrics unanimously recognized the importance of 5-aminolevulinic acid-based photodynamic therapy and reached a consensus on its clinical application in female lower genital tract diseases in 2022 (14). However, there are insufficient studies evaluating the efficacy of ALA-PDT for patients with cervical HSIL.

Our center, being one of the early adopters of ALA-PDT for treating female lower genital tract disorders, implemented this program in 2019. In this retrospective observational survey, we aimed to investigate the effectiveness and safety of ALA-PDT as a potential option for patients with cervical HSIL, as well as identify factors that impact its efficacy.

## 2 Materials and methods

### 2.1 Patients

The ALA-PDT treatment for female lower genital tract diseases was approved by the Ethics Committee at Hangzhou First people’s Hospital (Reference Number: 2021-010-01). A total of 40 patients with pathologically confirmed cervical HSIL who had undergone ALA-PDT at Hangzhou First people’s Hospital from Jan 2019 to Dec 2022 were enrolled in this study. Biopsies were taken under colposcopy guidance from the acetowhite and iodine unstained areas to obtain pathological tissues. All participants should have a strong desire to preserve the integrity and function of the cervix and voluntarily agreed to undergo ALA-PDT treatment with good compliance. Most of the patients selected had a pathologic diagnosis of CIN II. In our institution, we generally recommended surgical treatment for CIN III, unless the patient was fully informed and insisted on PDT. Histological results were assessed according to the 2014 World Health Organization Classification of female genital tumors (15): (1) normal, (2) low-grade squamous intraepithelial lesion, (3) high-grade squamous intraepithelial lesion (CIN II or CIN III), and (4) squamous cell carcinoma. All the enrolled patients had satisfactory colposcopy examination and their cervical transformation zones were required of entire visibility. Patients with type 3 transformation zones were not recommended for inclusion in the study. During the treatment and 3-month follow-up period, patients were required to abstain from intercourse. Informed consents were obtained from all participants before treatment. Patients with suspected invasive cancers or other kinds of lower tract disorders were excluded. Women in pregnancy and lactation should not be included in this study.

### 2.2 Cytological tests

Cytology tests were performed using a fully automated liquid-based cytology assay technology method (BD SurePath™) to evaluate abnormal cells. The cervical cytology results were then reported according to the Bethesda System 2014 (16).

### 2.3 HPV genotyping

HPV genotyping was performed using the PCR reverse dot hybridization method kit (Yaneng BIO science Co., Ltd., Shenzhen, China. REG. NO: CFDA 20233400811), to identify HPV infection with 17 high-risk types (HPV 16, 18, 31, 33, 35, 39, 45, 51, 52, 53, 56, 58, 59, 66, 68, 73, 82) and 6 low-risk types (HPV 6、11、42、43、81、83) (17).

### 2.4 5-ALA PDT procedure

All patients were placed in the lithotomy position, and then their vagina and cervix were cleaned by sterile 0.9% sodium chloride before treatment. Lesions of the cervix and cervical canal were completely covered with a sterile and thin cotton soaked in freshly prepared 20% 5-ALA solution (Fudan-Zhang Jiang Bio-Pharmaceutical Co., Ltd., Shanghai, China) for 3 hours. After cervical topical application of 5-ALA, a condom filled with medical gauze was inserted into the vagina to fix the cotton. Patients were then free to move during the waiting time.

**Figure 1:**
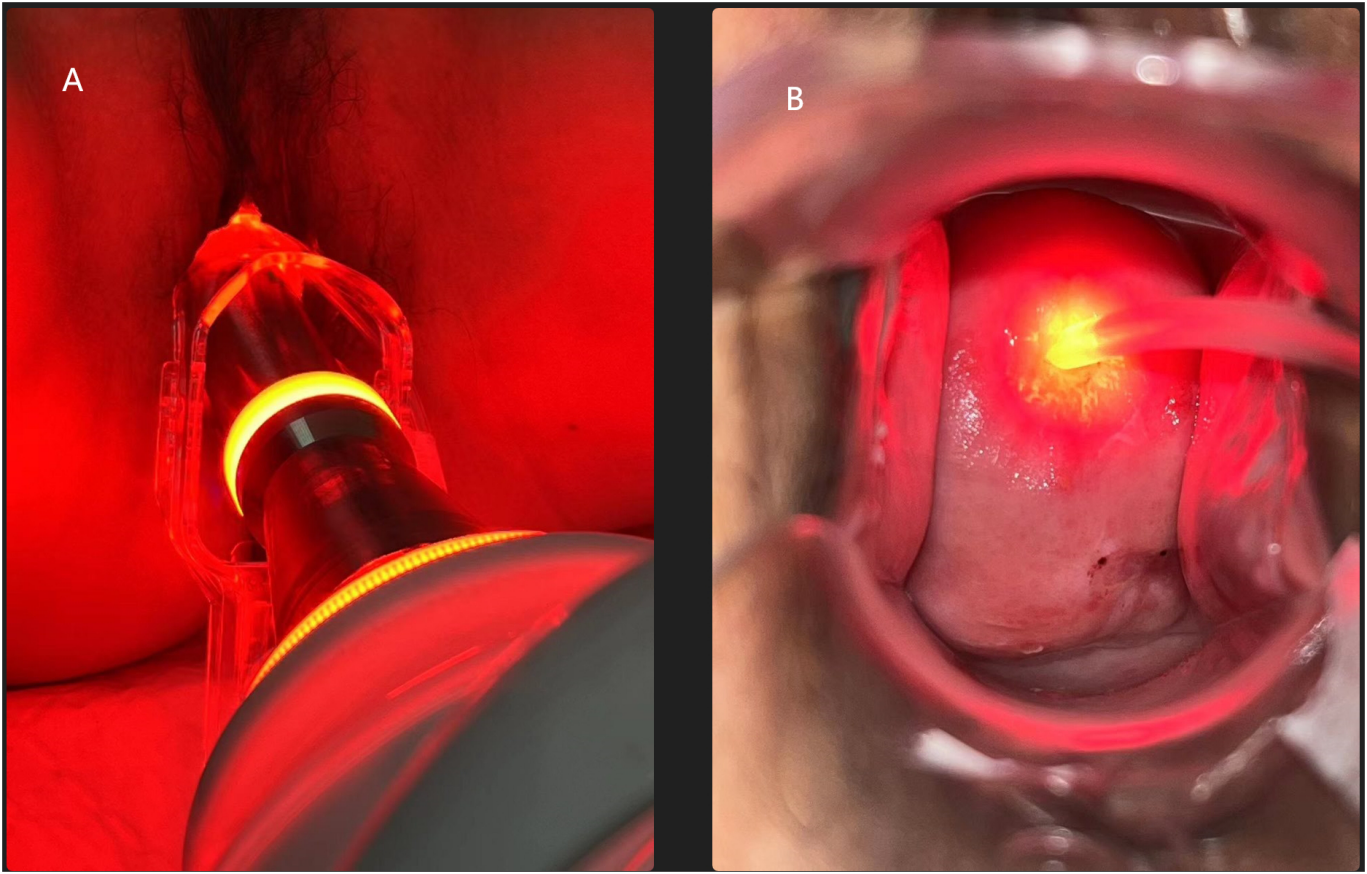
**(A)** An intravaginal light scattering cylindrical head applies to the cervical surface. **(B)** An optical fiber applies to the cervical canal.

Subsequently, a red light with an energy density of 80 mw/cm^2^ at a wavelength of 635 nm was applied to the cervical surface using an intravaginal light scattering cylindrical head (LED- IBS; Wuhan Yage Photo-Electronic Co., Ltd., Wuhan, China) (**Figure 1A**). Simultaneously, another red light was applied to the cervical canal using an optical fiber (LD600-C; Wuhan Yage Photo-Electronic Co., Ltd., Wuhan, China) (**Figure 1B**) for a duration of 30 minutes. The ALA-PDT procedure was scheduled 6 times for cervical HSIL patients. Interval between each time was one week, but postponed for one week during menstruation.

### 2.5 Follow up and clinical assessment

The treatment efficacy was evaluated by HPV genotyping and cervical cytology test at the 3-month follow-up post-treatment. If the HR-HPV turned negative and cytological result was normal, the treatment efficacy can be defined as complete remission (CR). If the cervical cytological result was worse than ASC -US, the patient should undergo colposcopy-directed biopsy immediately to evaluate the degree of cervical lesions, regardless of HR-HPV status. For other abnormalities, patients were advised to repeat HPV and cytology tests after 3 months, and if the abnormal results persisted or even progressed, timely colposcopy-guided biopsy is necessary to assess cervical lesions. ALA-PDT related symptoms and adverse events were also recorded.

### 2.6 Statistical analysis

The R software (Version 4.1.3) was used for data analysis. Chi-square and Fisher’s exact tests were performed to compare HPV clearance rates at 3-month follow-up after ALA-PDT for cervical HSIL among different groups based on various factors. Multiple Linear Regression was used to mitigate the impact of different variables. P value less than 0.05 was considered statistically significant.

## 3 Results

### 3.1 Clinical characteristics of the patients before treatment

A total of 40 patients with a pathological biopsy result of cervical HSIL were treated with ALA-PDT. Among them, 38 (38/40, 95%) had CIN II, and 2 (2/40, 5%) had CIN III. The average age of the patients was 28.25 years old (20 - 41 years old), and all of them wanted to preserve their cervical structure. Notably, among these patients, 26 were ≤ 30 years old, while 14 were older than 30 years. All patients were infected with HR-HPV before treatment. Among them, 23 patients were infected with subtype HR-HPV 16/18 and 17 patients were infected with other types. 27 patients had only one type of HR- HPV infection, while 13 patients had more than 2 types of HR-HPV infections. Before ALA-PDT, 19 patients had cytology evaluation ≥ LSIL, whereas 21 patients exhibited cytology evaluation < LSIL. 11 patients exhibited cervical canal lesions, while the remaining 29 patients had no cervical canal lesions. 24 patients had multiple sites lesions and the other 16 patients had only a single site lesion. The lesion involved the cervical glands in 10 patients. The detailed clinical characteristics of these patients are presented in **Table 1**.

**Table 1:** Baseline characteristics of the study population.

	Characteristics	Cases (n=40) %
Age	≤ 30 >30	26(65) 14(35)
Lesion grade	CIN II CIN III	38(95) 2(5)
HR-HPV subtype	HPV16/18 related Other types of HR-HPV	23(57.5) 17(42.5)
Number of HR-HPV	Only one More than two	27(67.5) 13(32.5)
Cytology	≥ LSIL < LSIL	19(47.5) 21(52.5)
Cervical canal lesions	Yes No	11(27.5) 29(72.5)
Multiple lesions		
	Yes	24(60)
	No	16(40)
Gland involvement		
	Yes	10(25)
	No	30(75)

### 3.2 The effect of photodynamic therapy in HSIL at follow-up

**Figure 2:**
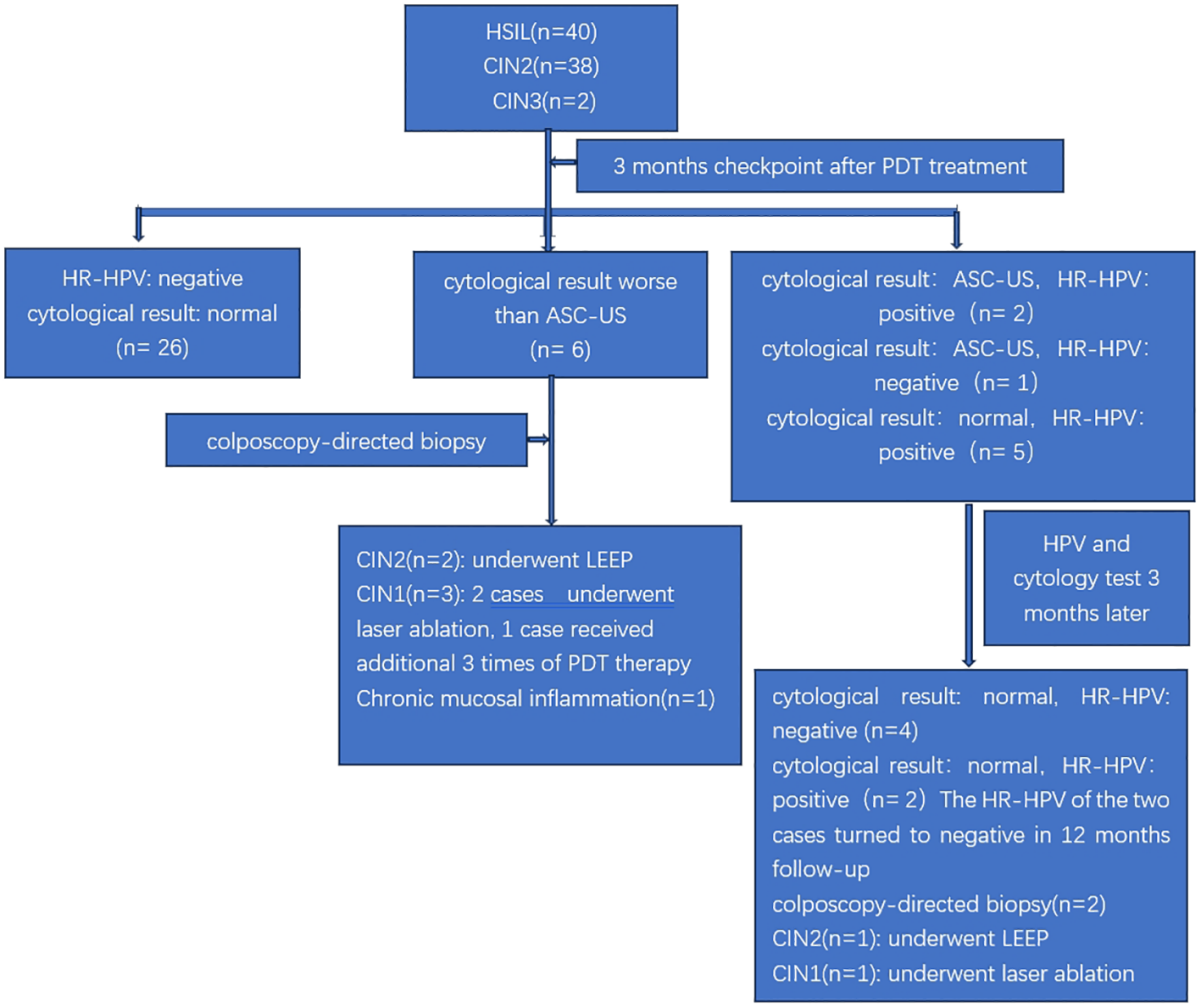
The follow-up flowchart of the study subjects.

The follow-up flowchart of the studied population is illustrated in **Figure 2**. All of the patients were evaluated for cervical cytological result and HR-HPV at the 3-month follow-up. Both cervical cytology and HR-HPV returned to normal in 26 out of the 40 cases. Therefore, the CR rate of ALA-PDT in cervical HSIL patients at our center was 65% (26/40) assessed at the 3-month follow-up. Six patients with cervical cytologic findings greater than ASC-US at the 3-month follow-up were directly referred for colposcopy-guided biopsy. The biopsy results indicated two cases of CIN II, which underwent LEEP; three cases of CIN I, of which two were treated with laser ablation and one received an additional course of PDT; and one case was confirmed to have chronic inflammation of the cervical mucosa. Additional details about these six patients were provided in **Table 2**. The remaining 8 patients had cervical cytology result of ASC-US and/or positive HR-HPV at the 3-month follow-up. In this group of patients, further evaluation was performed at 6 months after ALA-PDT to determine whether they should be referred for colposcopy-guided biopsy or kept under observation. Out of the eight patients, four had normal cervical cytology as well as negative HR-HPV at the 6-month assessment. Two patients had their cervical cytological and HR-HPV results normalized at the 12-month evaluation after ALA-PDT. Two patients underwent biopsy according to the 6-month evaluation, 1 was diagnosed with CIN II and underwent LEEP, while the other was diagnosed with CIN I and underwent laser ablation. The details on these 8 patients were provided in **Table 3**. Among the 38 patients with CIN II, 25 achieved CR after 3 months of treatment, with cervical cytology and HR-HPV completely turning negative. In addition, there were 6 CIN II patients who although did not achieve CR after 3 months of treatment, but 4 of them, and 2 of them, respectively, achieved CR at 6-month and 12-month follow-up. 81.6% (31/38)of CIN II patients achieved CR within one year of ALA-PDT in our study. Our study included two CIN III patients who strongly insisted ALA-PDT treatment. Their clinical and prognostic data are detailed in **Table 4**. One of the patients showed negative results in cervical cytology and HR-HPV infection after 3 months of treatment, achieving CR. Another patient was with cervical cytology indicating HSIL and HR-HPV persistent infection at the 3-month follow up. Further colposcopy biopsy revealed CIN II. The patient undergone LEEP surgery ultimately.

**Table 2:** Characteristics of the 6 patients with cytological result worse than ASC -US at 3-month follow up.

No	Age	Lesion Grade	Before PDT		GI	CI	Single lesion/ multicentric lesions	3-month follow up		Biopsy results	Further managements
			Cytology	HR-HPV				Cytology	HR-HPV		
1	20	CIN II	NILM	16+ other	No	No	multicentric lesions	LSIL	Other type	Chronic mucosal inflammation	Cytology and HR-HPV turned to be normal at 1-year follow-up
2	21	CIN II	ASC-US	16+ other	Yes	Yes	multicentric lesions	HSIL	16+ other	CIN II	LEEP
3	22	CIN II	LSIL	Other type	No	No	multicentric lesions	LSIL	Other type	CIN I	Laser ablation
4	24	CIN II	NILM	16	No	No	multicentric lesions	LSIL	Other type	CIN I	Laser ablation
5	27	CIN III	LSIL	Other type	Yes	Yes	Single lesion	HSIL	Other type	CIN II	LEEP
6	30	CIN II	NILM	16+ other	Yes	No	multicentric lesions	LSIL	16+ other	CIN I	Additional one course of PDT, Cytology and HR-HPV turned to be normal at 1-year follow-up

GI, gland involvement; CI, cervical involvement.

**Table 3:** Characteristics of the 8 patients with either cytological result ASC -US and/or HR-HPV positive at 3-month follow up.

No	Age	Lesion Grade	Before PDT		GI	CI	Single lesion/ multicentric lesions	3-month follow up		6-month follow up		Additional information
			Cytology	HR-HPV				Cytology	HR-HPV	Cytology	HR-HPV	
1	22	CIN II	LSIL	16	No	No	multicentric lesions	NILM	Other type	NILM	negative	
2	23	CIN II	LSIL	16	No	No	multicentric lesions	ASC-US	Other type	NILM	negative	
3	28	CIN II	NILM	16	Yes	Yes	Single lesion	NILM	16	NILM	negative	
4	30	CIN II	LSIL	Other type	No	No	multicentric lesions	NILM	Other type	NILM	Other type	Cytology and HR-HPV turned to be normal at 1-year follow-up
5	31	CIN II	ASC-H	16	No	Yes	multicentric lesions	NILM	16	NILM	16	Cytology and HR-HPV turned to be normal at 1-year follow-up
6	31	CIN II	ASC-H	Other type	No	No	multicentric lesions	ASC-US	negative	NILM	negative	
7	32	CIN II	ASC-US	Other type	Yes	Yes	multicentric lesions	ASC-US	Other type	LSIL	Other type	Further biopsy showed CIN II, LEEP was conducted
8	32	CIN II	LSIL	16+ other	No	Yes	multicentric lesions	NILM	16+other	NILM	16	Further biopsy showed CINI, laser ablation was conducted

GI, gland involvement; CI, cervical involvement.

**Table 4:** Characteristics and outcomes of the 2 patients diagnosed with CIN III.

No	Age	Lesion Grade	Before PDT		GI	CI	Single lesion/ multicentric lesions	3-month follow up		Biopsy results	Further managements
			Cytology	HR-HPV				Cytology	HR-HPV		
1	26	CIN II-III	NILM	16+	Yes	No	multicentric lesions	NILM	negative	/	Routine follow-up
2	27	CIN III	LSIL	Other type	Yes	Yes	Single lesion	HSIL	Other type	CIN II	LEEP

GI, gland involvement; CI, cervical involvement; NILM, No Intraepithelial Lesion or Malignancy; HSIL, high-grade squamous intraepithelial lesion; LSIL, low-grade squamous intraepithelial lesion; LEEP, Loop Electro surgical Excision Procedure.

In summary, none of the patients showed disease progression during the observation period after ALA-PDT. At the 3-month follow-up, 4 patients received other surgical methods because of incomplete remission of the disease on biopsy, and another 2 patients underwent surgical treatments at the 6-month follow-up for the same reason. Actually, among these six patients who underwent surgical treatment, four experienced partial lesion regression after ALA-PDT, while only two remained lesions that were consistent with pre-treatment. None of them experienced disease progression. The CR rate of ALA-PDT in cervical HSIL patients at our center was 65% (26/40) at the 3-month follow-up and reached 82.5% (33/40) at the 12-month follow-up.

### 3.3 Representative cases

**Figure 3:**
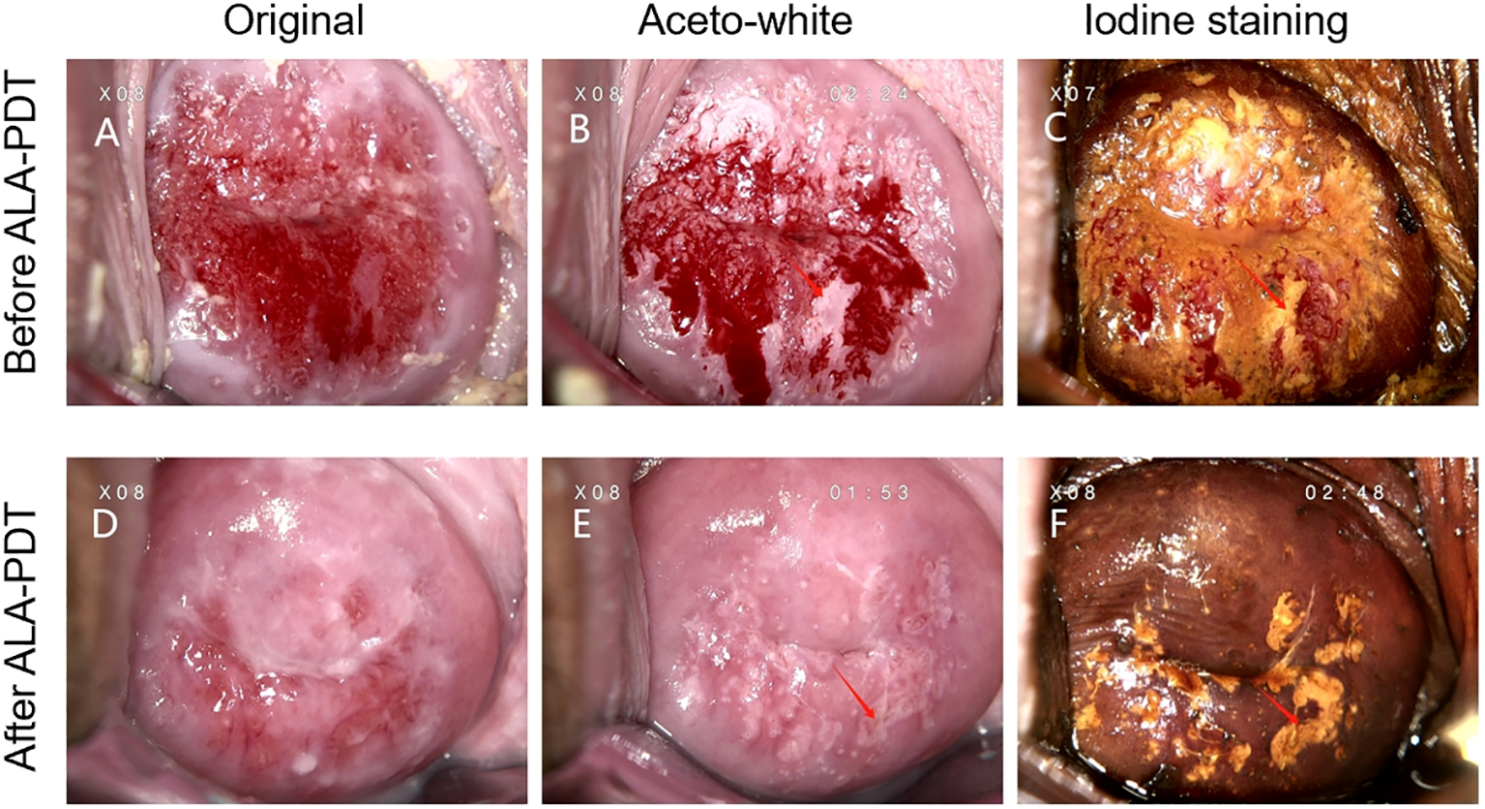
**(A–C)** Colposcopy images from case A who was diagnosed with CIN II before ALA-PDT. (**D–F)** Colposcopy images from case A who was diagnosed with cervical chronic mucosal inflammation 3 months after ALA-PDT treatment. The left column shows the initial performance of the cervical surface. The middle column depicts the aceto-white dysplastic lesion areas after the application of 3% acetic acid. The right column shows atypical epithelium after the use of iodine solution in the same patient. Arrows point to the appearances of the cervical high-grade squamous intraepithelial lesion before and after ALA-PDT.

Case A was a 20-year-old lady, G0P0, who had a history of abnormal vaginal bleeding after intercourse for 2 years. Her cervical cancer screening showed HPV 16,51 infection and normal cervical cytology result. The biopsy results of colposcopy indicated CIN II. Cervical cancer screening was repeated 3 months after the completion of 6 times of ALA-PDT, and the cervical cytological result showed LSIL and HPV 16 infection. The patient then received a repeated colposcopy-guided biopsy, which revealed chronic mucosal inflammation. Further observation found that her cytology and HR-HPV returned to normal at the 1-year follow-up. The colposcopy images before ALA-PDT and 3 months after ALA-PDT of this case are provided in **Figure 3**.

**Figure 4:**
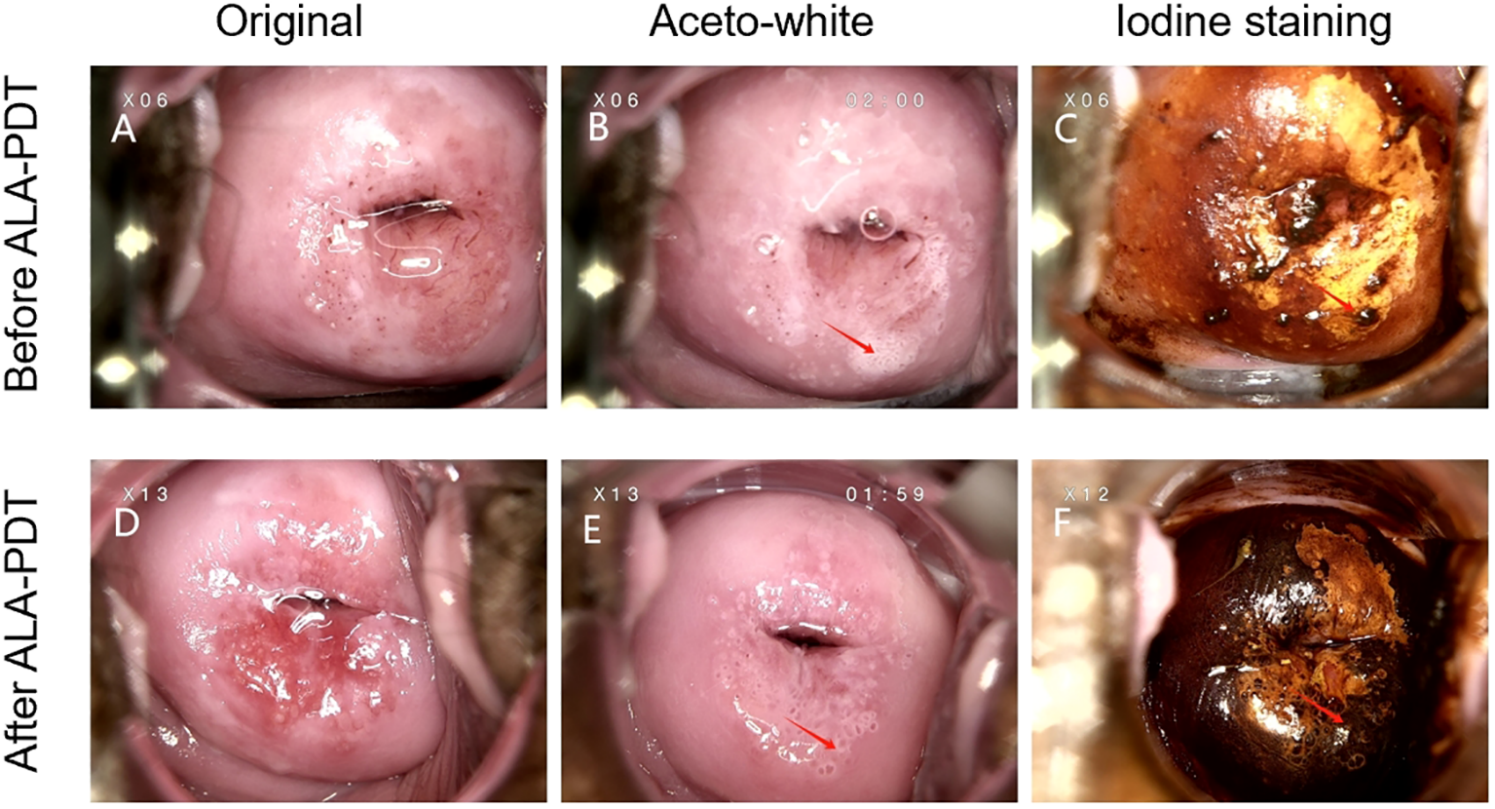
**(A–C)**. Colposcopy images from case B who was diagnosed with CIN II before ALA-PDT. **(D–F)** Colposcopy images from case B whose cervical cytology and HR-HPV turned to be normal 3 months after ALA-PDT treatment. The left column shows the initial performance of the cervical surface. The middle column depicts the aceto-white dysplastic lesion areas after the application of 3% acetic acid. The right column shows atypical epithelium after the use of iodine solution in the same patient. Arrows point to the appearances of the cervical high-grade squamous intraepithelial lesion before and after ALA-PDT.

Case B was a 24-year-old woman, G0P0, who was asymptomatic but tested positive for HPV 16,18,59,68 infection during a cervical screening. Her cervical cytological result was normal. The colposcopy biopsy results suggested CIN II with glandular involvement. The patient underwent six times of ALA-PDT treatment, and a repeated cervical screening 3 months later showed the disappearance of HR-HPV and normal cervical cytology. Meanwhile, colposcopy images before and 3 months after ALA-PDT of this case were provided in **Figure 4**.

### 3.4 Factors affecting the rate of HPV clearance 3 months after PDT

**Figure 5:**
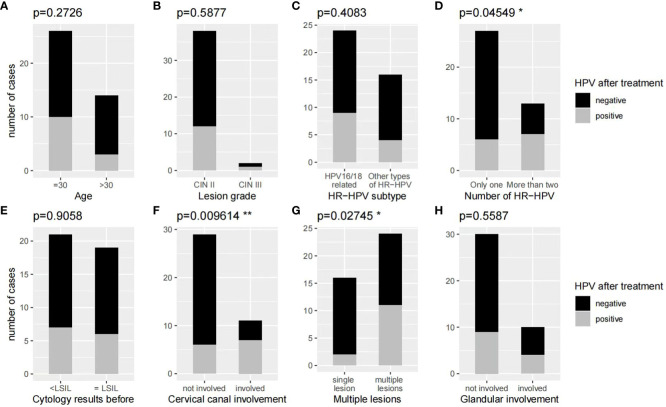
**(A, B, C, E, H)** Univariate analyses results showed that age, lesion grade, HR-HPV subtype, cytology, and cervical gland involvement before treatment of ALA-PDT have no effect on the HR-HPV clearance rate at the 3-month follow-up. **(D, F, G)** Univariate analyses results showed that cervical canal involvement, cervical multiple sites lesions, and more than one type of HR-HPV infection are risk factors for persistent HR-HPV infection at the 3-month follow-up.

We found that the persistent HR-HPV infection risk was 32.5% (13/40) in the 3-month follow-up. To evaluate the factors influencing the rate of HPV clearance 3 months after ALA-PDT, we conduct both univariate and multivariate analyses on age, lesion grade, HPV subtypes, number of HPV types, cervical cytological results before treatment, cervical involvement, glandular involvement, as well as the number of lesions. Univariate analyses results showed that the HPV clearance rate was significantly lower in patients with cervical canal involvement (36.36% vs 79.31%, p<0.05), multiple lesions (54.17% vs 87.5%, p<0.05) and infection with more than one species of HPV (46.15% vs 77.78%, p<0.05) when compared to those without cervical canal or glandular involvement. The analysis revealed that factors such as age, lesion grade, HPV subtype, cytology results before treatment, and number of lesions before ALA-PDT have no impact on the HPV clearance rate (**Figure 5**). However, after multivariate regression analyses, we found that cervical canal involvement was the only independent risk factors affecting the clearance rate of HPV 3 months after completion of PDT (**Table 5**).

**Table 5:** Multiple linear regression analysis of HPV clearance rate after ALA-PDT.

	Ratio of HPV positive patients		Effect on HPV Clearance (P Value)
Age	<=30	10/26	0.1823
	>30	3/14	
Lesion grade	CIN II	12/38	0.9848
	CIN III	1/2	
HR-HPV subtype	HPV16/18 related	9/24	0.6338
	Other types of HR-HPV	4/16	
Number of HR-HPV	Only one	6/27	0.1525
	More than two	7/13	
Cytology	≥ LSIL	6/19	0.2858
	< LSIL	7/21	
Cervical canal lesions	Yes	7/11	0.0066^*^
	No	6/29	
Multiple lesions	Yes	11/24	0.0537
	No	2/16	
Gland involvement	Yes	4/10	0.4994
	No	9/30	
Total	13/40		0.0248^*^

Values with P<0.05 are marked in bold, which mean significant difference between groups.

### 3.5 Incidence of side effects

No patients experienced severe side effects on vital signs such as blood pressure, heart rate and breath. Most patients were observed mild local adverse effects during of a few days after ALA-PDT. These effects included increased vaginal discharge, slight abnormal pain, slight vaginal bleeding and burning sensations. Fortunately, these side effects were bearable and relieved within one-week post-treatment. Notably, some patients were observed to have mental disorders such as anxiety or insomnia during the follow-up period. This may be attributed to the fear of the disease and concerns about the efficacy of the treatment.

## 4 Discussion

Currently, cervical cancer is the fourth most common malignancy in women worldwide, and it is still a major cause of cancer-related death in some of the world’s poorest countries (18). In 2018, the World Health Organization issued a global call for the elimination of cervical cancer as a public health problem (19). The strategies for eliminating cervical cancer included primary prevention via HPV vaccination, secondary prevention via cervical screening, and the third prevention of timely management of precancerous lesions. HSIL of the cervix, which is induced by HR-HPV, is a premalignant disease. In the past, surgical excision using methods like cold knife conization, laser conization/ablation, or LEEP was the gold standard treatment for cervical HSIL (4). Because of young women’s low-acceptance of surgical procedures and the associated complications, as well as their strong desire for complete preservation of cervical tissues, it is imperative to explore a non-invasive and effective method for the management of cervical HSIL. ALA-PDT seems to be a good choice for this specific group of patients.

ALA-PDT involves the selective accumulation of 5-aminolevulinic acid in the CIN tissues (20), which are then illuminated to generate ROS that destroy tumor cells by inducing apoptosis and necrosis. The success of the process relies on three key points: oxygen-induced activation of photosensitizer, appropriate utilization of visible light, and proper selection of the photosensitizer (21). This process is highly tissue selective, non -invasive, and carries a low risk of severe complications, making it an ideal method for treating cervical HSIL, especially for young women. Compared to traditional surgical excisions, ALA-PDT is characterized by elimination of precancerous lesions and potential HPV infection (22) without causing damage to the normal anatomy.

Our study showed that ALA-PDT was highly effective in treating a cervical HSIL, leading to a complete remission rate of 65% at 3 months after completion of ALA-PDT. Furthermore, with an extended observation period, the complete remission rate could reach as high as 82.5% at 12 months after the completion of ALA-PDT. These findings are consistent with previous studies. Wu et al. reported a histological complete remission rate of 77.78% for CIN II after PDT at 12-month follow-up (23). Tang et al. revealed that the cervical HSIL complete remission rate after PDT was 88.9%, 92.5% at 6-months,12-months follow-up respectively (24). Qu et al. found that the total lesion regression rate of cervical HSIL after PDT was 89.58% at 3-months follow-up (25). Hu et al. reported that the disappearance rate of cervical HSIL after PDT was 81.82%, 90.91% at 3-month,6-month follow-up respectively (26). Although the above studies reported a satisfactory efficacy of PDT for HSIL treatment, there are still some limitations. The application of ALA-PDT in cervical HSIL is still in the initial exploration stage, and the included cases in the above studies were limited, which might limit the generalizability and robustness of the conclusions. Further studies with high-level of evidence, such as multicenter, large-sample, randomized controlled clinical trials, are necessary to be conducted to validate our findings. Additionally, it should be noted that our study primarily focused on patients with CIN II, only 2 patients with CIN III were included. One patient was diagnosed with CIN II-III and responded positively to ALA-PDT treatment, showing normal cervical cytological and HPV test results after three months. Conversely, the second patient, diagnosed with CIN III, did not benefit from this therapy and instead achieved recovery through LEEP. This discrepancy in treatment efficacy may be attributed to the greater lesion depth associated with CIN III, which renders non-invasive treatments such as ALA-PDT less effective than they are for CIN I and CIN II cases. Due to the lack of sufficient data, we cannot make some rigorous suggestions for CIN III patients. Further research including larger subjects is needed to demonstrate the effectiveness of PDT for patients with CIN III.

There is no consensus on the follow-up strategy after PDT. In our study, we evaluate the combination of HPV genotyping and cervical cytological results as an initial evaluation at the 3-month follow-up. Cases were then triaged according to the results. The stratification protocol in our study was to immediately refer to colposcopy-guided biopsy if the cytologic result was worse than ASC-US at the 3-month follow-up. If the cervical cytological result was ASC-US and/or there was HR-HPV infection, observation could continue with repeated cervical screening at 6-months follow-up. We designed this stratification protocol based on previous studies reporting that PDT could enhance local and systemic immunity, which plays a crucial role in clearing lesions (27–29). Therefore, we have implemented a relatively conservative observation protocol for patients with either cytology result ASC-US or HR -HPV infection 3 months after PDT treatment. We hypothesized that the activated immune response by PDT would have a long-term effect on the clearance of lesions or HR-HPV, thus ensuring the safety of this group of patients. In our study, 8 cases that met this criterion(either cytologic result ASC-US and/or HR-HPV infection). Among them, 6 patients returned to normal cytology and negative HPV within 1 year by observation, one patient regressed from CIN II to CINI, and one patient persisted in CIN II. None of the patients had a history of disease progression during the course of observation. Therefore, we consider our triage program to be reasonable.

In our study of single factor analysis, we found that cervical canal involvement, multiple lesions and infection with more than one species of HR-HPV can cause persistent viral infection at the 3-month follow-up after ALA-PDT treatment. However, multiple linear regression analysis revealed that only cervical canal involvement was an independent risk factor. These findings suggest that the effects of multiple lesions and HR-HPV species are secondary to the difference in cervical canal involvement. Consequently, even if a patient has numerous lesions and HR-HPV infection, if there is no disease in the cervical canal, ALA-PDT can effectively eliminate her HR-HPV infection. We infer that the reason maybe the optical fiber is not as efficient as the intravaginal light scattering cylindrical head. Thus, we recommend to prolong the time of irradiation by the optical fiber for patients with cervical canal lesions. The underlying association between cervical involvement and persistent HR-HPV infection requires further research. It is important to monitor women with persistence of HR-HPV infection who have underwent PDT therapy as it greatly impacts the prognosis. Our findings emphasize the need to pay extra attention to the patients with cervical involvement.

During the treatment of the 40 patients with ALA-PDT, there were no severe adverse reactions. A few patients presented with discomfort symptoms, such as burning sensations, slight pain, slight discomforts in the lower abdomen and increased vaginal discharge. These adverse reactions were bearable and were relieved within a few days after treatment, indicating the safety of ALA-PDT.

During our research, patients underwent a treatment regimen of ALA- PDT with a frequency of once per week, typically completing six sessions. The entire course of treatment spanned approximately 7 weeks to 2 months, which is notably longer than the single-session requirement of other non-invasive therapies such as laser, cryotherapy, and thermal ablation. Despite the extended duration, ALA-PDT offers the advantage of being less painful in comparison to these alternatives. Moreover, the satisfactory outcomes observed in this study can be partly attributed to the regularity of the ALA-PDT treatment sessions.

Non-invasive physical plasma (NIPP), which inhibit pathological cell growth through rapid and transient DNA damage, is another emerging approach for cervical precancerous lesions. Like ALA-PDT, NIPP is a tissue-preserving and easy-to-apply method, which can be performed in outpatient settings without the need for local or general anesthesia and is more cost-effective than ALA-PDT. Previous studies have reported that the complete remission rate of NIPP to CINI/II reached 86.2% -95% at 3-6 months after treatment (30, 31). However, these studies were single arm prospective studies and included a small sample size. Further studies are expected to include larger population and reveal the efficacy of NIPP in CINII/III patients. NIPP may be an underlying alternative to ALA-PDT for the treatment of cervical HSIL.

## 5 Conclusion

1. ALA-PDT is a highly effective, non-invasive, and safe therapeutic intervention for cervical HSIL. Compare to surgical procedures, it has the advantage of preserving the structural and functional integrity of the cervix. Therefore, it is an optimal choice for young women with cervical HSIL who have fertility requirements. 2. We suggest continuous observation instead of performing a colposcopy-guided biopsy in patients who have either a cervical cytological result of ASU-US and/or a HR-HPV infection at 3-month follow-up after ALA-PDT therapy. 3. Cervical canal involvement is an independent risk factor for persistent HR-HPV infection at the 3-month follow-up after PDT treatment.

## Data availability statement

The original contributions presented in the study are included in the article/supplementary material. Further inquiries can be directed to the corresponding authors.

## Ethics statement

The studies involving humans were approved by Ethics Committee of Hangzhou First people’s Hospital. The studies were conducted in accordance with the local legislation and institutional requirements. The participants provided their written informed consent to participate in this study.

## Author contributions

JQ: Writing – review & editing, Writing – original draft, Formal analysis, Data curation, Conceptualization. YW: Writing – review & editing, Supervision, Investigation. GW: Writing – review & editing, Resources, Data curation. JL: Writing – review & editing, Resources. LS: Writing – review & editing, Supervision, Conceptualization. SX: Writing – review & editing, Software, Methodology, Formal analysis, Conceptualization.
